# Network of microRNAs-mRNAs Interactions in Pancreatic Cancer

**DOI:** 10.1155/2014/534821

**Published:** 2014-05-07

**Authors:** Elnaz Naderi, Mehdi Mostafaei, Akram Pourshams, Ashraf Mohamadkhani

**Affiliations:** ^1^Liver and Pancreatobiliary Diseases Research Center, Digestive Diseases Research Institute, Tehran University of Medical Sciences, Tehran, Iran; ^2^Biotechnology Engineering, Islamic Azad University,Tehran North Branch, Tehran, Iran

## Abstract

*Background.* MicroRNAs are small RNA molecules that regulate the expression of certain genes through interaction with mRNA targets and are mainly involved in human cancer. This study was conducted to make the network of miRNAs-mRNAs interactions in pancreatic cancer as the fourth leading cause of cancer death. *Methods.* 56 miRNAs that were exclusively expressed and 1176 genes that were downregulated or silenced in pancreas cancer were extracted from beforehand investigations. MiRNA–mRNA interactions data analysis and related networks were explored using MAGIA tool and Cytoscape 3 software. Functional annotations of candidate genes in pancreatic cancer were identified by DAVID annotation tool. *Results.* This network is made of 217 nodes for mRNA, 15 nodes for miRNA, and 241 edges that show 241 regulations between 15 miRNAs and 217 target genes. The miR-24 was the most significantly powerful miRNA that regulated series of important genes. ACVR2B, GFRA1, and MTHFR were significant target genes were that downregulated. *Conclusion.* Although the collected previous data seems to be a treasure trove, there was no study simultaneous to analysis of miRNAs and mRNAs interaction. Network of miRNA-mRNA interactions will help to corroborate experimental remarks and could be used to refine miRNA target predictions for developing new therapeutic approaches.

## 1. Introduction

Pancreatic cancer has introduced itself as one of the top five causes of cancer mortality. Although many efforts have been attempted, the prognosis is still not desirable in which surgical resection has been remained as the last choice for the patients with advanced stage of disease [1]. It is worthy of mention that even surgery has not made a great step in mortality (improving 5-year survival rates from <4% if left untreated to 25–30% after resection) [2]. As seen in other cancers, pancreatic cancer also occurs due to genetic alterations and disturbances of gene expression [3]. One of regulatory-genetic factors that have been identified in the past decade is MicroRNAs (miRNAs). The miRNAs with 20–22 nucleotides (nt) in length are a group of small noncoding RNA molecules with considerate function in posttranscriptional phase [4]. Modulation of apoptosis and expression of genes involved in invasive behavior indicate the role of miRNAs in cancer. Depending on the cell, tissue, and disease type, the expression pattern of miRNAs could be different.

Today, it has been proved that deregulation in expression of miRNAs has important role in the pathogenesis of genetic and multifactorial cancers, as well as pancreatic cancer [5, 6]. Because the majority of human protein-coding genes could be regulated by miRNAs, equally it was assumed that miRNAs could have several target genes [7] though miRNAs are apt to downregulate target mRNAs [8]. These findings suggested that nearly more than one-third of human genes are regulated by miRNAs through various silencing pathways. Bearing in mind complex gene regulatory networks in biological systems, many scientific efforts have been devoted to draw “the big picture” in miRNA-mRNA interactions using the combination of experimental methods and computational approaches. Most often disregulation of miRNAs has reflected the pathophysiology of many cancers such as liver, prostate, and ovarian [9–11]. Moreover, molecular disregulation of miRNAs is believed to play major roles in etiology of pancreatic cancer or be a consequence of tumor formation that could be used as a biomarker for tumor detection. Indeed expression of miRNAs correlated with outcome in pancreatic cancer patients [12–15]. Recognizing mRNAs regulated by miRNAs will help us for better understanding of biological roles of miRNAs [16]. Although the collected previous data seem to be a treasure trove, a large portion of data is false-positive or insignificant. Many computational methods have been developed to resolve this problem to extract precise results. In this way, researches on miRNAs-mRNAs interactions can lead us to pathways from their identification to their functional assignment in system biology. One motivation of systems biology research is to understand gene functions and up-to-date structural and functional annotations of genes [17, 18].

Pancreatic cancer is often diagnosed at a late stage and surgical resection, if possible, is too difficult. Recognizing early diagnosis biomarkers and molecular targets therapy need to discover that was one important aim in experimental studies [19, 20]. Furthermore, investigation of miRNAs-mRNAs interactions in this cancer would be new approach to gaining insight into complex biological processes not only for brighter understanding of pancreatic cancer etiology but also for designing new prognostic and diagnostic strategies. In this study, we performed a number of available software programs for the purpose of construct of most efficient miRNA-mRNA network analysis and visualization.

## 2. Methods

### 2.1. Search Studies and Datasets

PC-related miRNAs were collected from miR2Disease base (http://www.mir2disease.org/) database and also from the relevant literature. There were a total of 64 pancreatic cancer miRNAs, in which, 8 miRNAs were downregulated and 56 miRNAs upregulated (Table 1). Overall upregulated miRNAs which have been described as oncogenes were selected for miRNA-mRNA network analysis. The same strategy was performed for mRNA expression pattern in PC. Gene Expression Omnibus (GEO; http://www.ncbi.nlm.nih.gov/geo), ArrayExpress (http://www.ebi.ac.uk/arrayexpress/), Stanford Microarray Database (http://smd.princeton.edu/), and PubMed (http://www.ncbi.nlm.nih.gov/pubmed) are comprehensive databases for gene expression.

### 2.2. miRNA-mRNA Interaction Analysis

In this study, information of mRNAs and miRNAs expression profiles retrieved from different molecular methods; therefore we integrated the data of miRNA and gene expression as not matched samples by MAGIA tool (http://gencomp.bio.unipd.it/magia) because matched data (samples have been produced in a lab) were not available. Not matched data analysis option in MAGIA tool is functional for samples in public database. The data collection and processing give rise to two matrices, for miRNAs and genes. Each matrix includes two classes of samples (T for tumor and N for normal), for the miRNA and gene matrices. The meta-analysis separately considers each matrix to identify expression profiles significantly variable among classes and integrate results with target predictions. Note that expression matrices must be tab-delimited text files; the first row must contain sample names; the first column must contain gene/miRNA IDs.

### 2.3. Integrative Analysis with Not Matched Expression Matrices in Multistep Procedure

1. We selected ID type (RefSeq) and “meta-analysis” from the list of available methods for the integrated analysis. MAGIA will calculate LIMMA *P* values of differential expression, which are then combined by using the inverse chi-square distribution to identify oppositely variable miRNA-gene pairs.
2. Select a target prediction algorithm (miRanda, PITA, and TargetScan) or a combination thereof. In this study to ensure more, RefSeq ID and the intersection of all three algorithms were used.
3. Upload miRNAs and genes expression matrices.
4. Finally, construction of network was performed via the sequence complementarity between miRNA and mRNA. Functional enrichment analysis was directly uploaded, with corresponding settings on the DAVID page. Moreover for network visualization a text file was imported in Cytoscape software. The Evolutionary Trace Annotation (ETA) method in the Cytoscape network visualization environment was carried out for predicting the functional associations among miRNAs and mRNAs [31]. This software supports the visual features of nodes and edges, such as shape, color, and size. In this network length of edges are proportional to the log *P* value, therefore genes that are located on the farther representing by smaller amount of *P* value.

## 3. Gene Ontology and Pathway Analysis 

The Sequence Retrieval System GeneDecks (http://www.genecards.org/genedecks) that are a novel analysis tool for transcriptome analyses and comprise the largest databases of genes were used to establish the targeted gene lists. Furthermore, DAVID Bioinformatics Resources 6.7 (http://david.abcc.ncifcrf.gov/) were used for understanding the biological meaning behind large lists of genes, to obtain gene ontology and pathway information for significant downregulated target genes in pancreatic cancer. The important functions and biological roles of target genes and their association with pancreatic cancer were assessed by MALACARD server (http://www.malacards.org/).

## 4. Results

### 4.1. miRNAs and mRNA Expression Profiling

Overall 64 pancreatic cancer miRNAs, in which 8 miRNAs were downregulated and 56 miRNAs upregulated, have been identified from databases. The 8 downregulated miRNAs were not enough for meta-analysis by MAGIA; therefore they have been excluded from network analysis. By the way upregulated miRNAs which have been described as oncogenes are more suitable for gene therapy and considered as biomarkers. Expression profile of most significantly overrepresented miRNAs that were used for miRNAs matrices in pancreatic cancer was indicated in Table 1. There were also 1176 genes from some overlap data to deviated outlying data, with lower expression compared to normal condition. As miRNAs tend to downregulate target mRNAs, these types of genes entered into mRNA matrices. Note that information of each matrix includes normal condition and pancreatic cancer condition. After analyzing and matching matrices by varied algorithms, it was determined that many of miRNAs and mRNAs had no target and matching *P* value was more than 0.05 in many of them. Finally, 15 types from 56 miRNAs and 217 types of target genes, whose *P* values were less than 0.05, were placed in the network.

### 4.2. miRNA-mRNA Network Analysis

**Figure 1:**
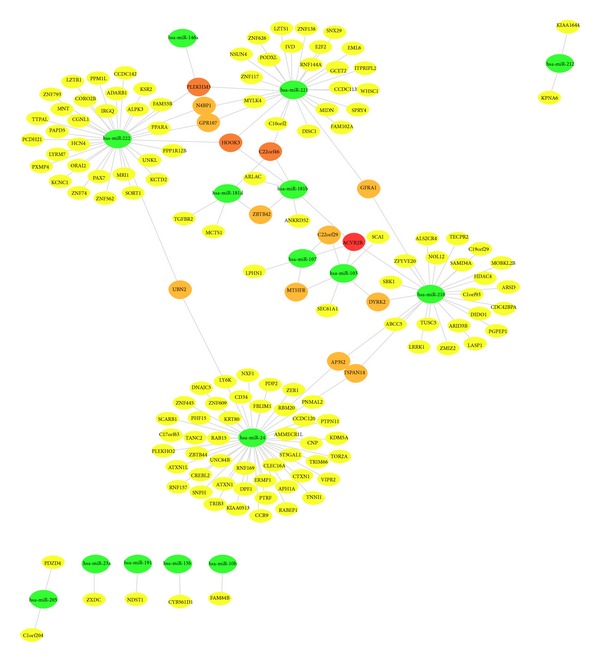
Interaction network of mRNAs and upregulated miRNAs in pancreatic cancer based on Cytoscape 3 software. Green circles shower miRNAs and other circles offer for mRNAs that are existing in yellow, orange, brick red, and red based on the number of their regulating miRNAs. When the colors tend to be red from yellow, the numbers of interactions increase. Yellow color represents one interaction. It means mRNA is controlled and regulated by miRNA. Orange color represents two interactions; brick red color, three interactions; and red color, four interactions and regulate by four miRNAs. Length edges are log *P* value. (If you zoom in the figure, you will find more details regarding network.)

Figure 1 shows the miRNA-mRNA interaction network and mRNA targets of differentially expressed miRNAs in pancreatic cancer that have been evaluated by gene networking tools (MAGIA and Cytoscape). This network consists of 217 nodes for mRNA, 15 nodes for miRNA, and 241 edges that show 241 regulations between 15 miRNAs and 217 genes. The miRNAs are presented in green circle while other circles offer for mRNAs that are existing in yellow, orange, brick red, and red based on the number of their regulating miRNAs. In this network, all edges of miRNAs (green circle) tend to mRNAs (circles by other colors), because miRNAs regulate mRNAs. If you zoom in Figure 1, you will find more details regarding network. Generally, when the colors tend to be red from yellow, the numbers of interactions increase. Yellow color represents one interaction while orange color represents two; brick red color, three; and red color, four interactions. On the other hand, *P* values of some of mRNA, which were assigned to their interaction with miRNAs, were more than 0.05. Therefore, they did not enter network. According to this network 197 target genes from 217 are regulate by four miRNAs.

### 4.3. Target Gene Analysis in Pancreatic Cancer

GeneCards is a searchable, combined, database of human genes that provides concise genomic related information, on all known and expected human genes. A portion of the GeneCards is related to the “Gene Associated with Disease” that represents proven roles of each gene in various diseases based on studies that have been done to date. GeneCards information displayed 20 of 217 expressional genes in interaction with fifteen miRNAs in the network which have had a permanent role in cancer of pancreas. We also have benefited Malacard site to understand how much every 20 genes are affective. Malacard score is sign of relationship of this gene to pancreas cancer that are presented in Table 2. Represented score in this site equals the rank of GenDecks *P* value and gene-associated score, multiple by the product of the logs of the ranks in gene cards search hit scores for each of genes. In addition to these 20 genes, the roles of 54 genes in this network are approved in other cancers especially gastrointestinal cancer. Therefore, 74 genes of 217 genes in the network are approved genes regarding cancer.

Moreover, mRNAs that are targeted with more than one miRNA are listed in Table 3. This list describes some of the most notable genes and interactions between miRNAs and target genes in pancreatic cancer. According to these findings, our network revealed that ACVR2B, GFRA1, and MTHFR are the most gene transcripts that are regulated by miRNAs and their relationships to pancreatic cancer were confirmed previously. The importance of mentioned three target genes rather than others is that they are shared in Tables 2 and 3. These findings revealed that genes which are known specifically for cancer of pancreas (Table 2) are regulated by more than one miRNA under pancreas cancer condition (Table 3). In other words, there is a specific control on expression of these three genes in deployment of pancreatic cancer. There are other genes in this network that are controlled and regulated by more than one miRNA (Table 3). However, their role has not been studies clearly in pancreatic cancer. In addition, miR-24, miR-210, miR-221, and miR-222 are the most important among miRNAs. According to Figure 1, many of existing mRNAs in the networks are controlled and regulated by these four miRNAs. The important one is miR-24. Because many of target genes represented in Tables 2 and 3 are controlled and regulated by this type of miRNA.

## 5. Gene Ontology Annotation

GO annotation strategies also permit the functional annotation of candidate genes that were uniquely downregulated in pancreatic cancer. In Table 4, gene ontology (GO) annotations for predicted target mRNAs in miRNA-mRNA interaction network were determined by DAVID and GeneDecks. In view of phenotype, the miRNA target genes of the current network are organized in three groups mortality/aging (50 genes), growth/size (42 genes), and behavior/neurological (36 genes). Genes were predominantly grouped into functional classes of protein binding (63 genes), sequence-specific DNA binding transcription factor activity (19 genes), and zinc ion binding (20 genes) (Table 4). The most important biological processes are transcription, DNA-dependent, and smoothened signaling pathway. Cellular constituents of genes mainly belong to the cytoplasm, nucleolus, and nucleus. This term denote the place of activity of a gene product. The evolutionary domains of miRNAs target genes products in the presented network are Ser/Thr-kinase-AS, PHF, Prot-kinase-cat-dom, Znf-PHD-finger, Kinase-like-dom, and Znf-PHD.

## 6. Discussion

In our study, we used system biology tools and high-throughput experiment data (expression profiles mRNA/miRNA from literatures) to analyze the interaction of miRNAs with mRNAs that are targeted by them and are involved in cancer of pancreas. In complex diseases like cancer the function of genes deregulate in overlapping pathways. Tumor cells use preexisting pathways in altered ways and they combine components of these pathways in an innovative way. The relevance of miRNAs in pancreatic cancer and its therapy is now under intense investigation [12, 32]. Individual miRNAs can target multiple mRNAs genes that are involved in self-organization and homeostasis of living organisms. Analyzing the network of gene-expression data make known the organizational of gene expression and recognize new potential drug targets in cancer.

This study has determined that 197 of 217 genes are targeted and downregulated by only four of the miRNAs: miR-24, miR-210, miR-221, and miR-222 in which miR-24 was the most important because numerous target genes, represented in Tables 2 and 3, are controlled and regulated by miR-24. This miRNA suppresses the expression of tumor suppressor genes and cell cycle control genes by targeting the 3′-UTR of their mRNA [33]. Moreover miR-210, miR-221, and miR-222 were significantly proficient to regulate more than one target gene which were associated with pancreatic cancer in this network. Remarkably, many of miRNAs which in previous studies were significantly and specifically determined had no target in this network. Indeed, large amount of related genes to pancreatic cancer in published studies not to be found in this network revealed they are not regulated by any of 56 types of candidate miRNAs. MTHFR, GFRA1, and ACVR2B were recognized as the most important controlled genes in present network. We showed that ACVR2B (Activin A receptor, type IIB) gene linked to four types of miRNA and regulated many physiologic and pathobiology processes including immunosuppression, carcinogenesis, insulin secretion, and pancreas development [34]. The expression pattern and mutations of ACVR2B has been approved in previous studies [34, 35]. Moreover, GFRA1 (GDNF family receptor alpha-1) is a potent neurotrophic factor that plays a key role in the control of neuron survival and differentiation. Somewhere, this gene is introduced as a function of age [36]. Also, MTHFR (methylenetetrahydrofolate reductase) was proposed to participate in metabolic pathway. One carbon pool is provided by foliate implicated in blood circulation, cellular amino acid metabolic process, methionine biosynthetic process, and oxidation reduction [37]. Association of these target genes with the pathophysiology of pancreatic cancer has been already validated [32, 38]. Indeed their functional roles are explained by biological process, molecular function, and cellular component GO annotations. In eukaryotic cells, GO annotations of related proteins to target genes revealed several processes through their domains that carry out diverse molecular functions and participate in multiple alternative interactions with other proteins, organelles, or locations in the cell (Table 4).

We confess that many of obtained outputs of high-throughput experiments do not exist in this network anymore. The reason of such different gene expression profiles from experimental studies of pancreatic cancer is that there was no study simultaneous to analysis of miRNAs and mRNAs in the same condition. Moreover, considering cognitive world of transcriptomic and regulating factors, it is not expected that miRNAs act just as regulator of mRNAs, because new findings show that target of some miRNAs is long noncoding RNA (lncRNAs). In addition, expressions of many RNAs are controlled by other regulating factors instead of miRNAs. However, according to this matter that more than 60% of expressions of protein producer genes in human are regulated by miRNAs and this fact that the more important and famous regulating mechanism for expression genes are miRNAs, one of scientists' purposes is studying and controlling them under different circumstances.

## 7. Conclusion

Previous works investigated just expression of miRNAs or mRNAs related to pancreatic cancer that are a rich treasury of information. They are possible to achieve to biomarkers. However, by drawing connecting networks, it leads us to the main passkeys in regard to the relationship between miRNAs and mRNAs in occurrence or development of cancer under certain conditions. We demonstrate bioinformatics approach to study the key communication between mRNAs and miRNAs. The obtained results of this network revealed that 217 mRNA were significantly regulated by 15 miRNAs in pancreatic cancer. The miR-24 has been reported as most important miRNA and genes such as ACVR2B, GFRA1, and MTHFR are most involved in pancreatic cancer. Therefore this study provides new opportunities for studying novel molecular pathways of pancreatic cancer pathogenesis and for developing new therapeutic approaches.

## Figures and Tables

**Table 1:** Designated studies for expression profile of microRNAs in pancreatic cancer.

Number	References	Detection method	microRNAs
1	[21]	Northern blot, qRT-PCR	hsa-let-7f-1, has-let-7d, miR-21, miR-212, miR-16-1, miR-424, miR-92-1, miR-107, miR-181c, miR-15b, miR-155, miR-24-1, miR-24-2, miR-301, miR-376a, miR-181a, miR-221, miR-100, and miR-125-1
2	[22]	Microarray	miR-205, miR-99a, miR-21, miR-210, miR-213, miR-107, miR-10a, miR-10b, miR-181b-1, miR-181b-2, miR-181c, miR-181d, miR-143, miR-199a-1, miR-199a-2, miR-155, miR-222, miR-223, miR-23a, miR-23b, miR-181a, miR-220, miR-221, miR-100-1, miR-100-2, miR-103-2, miR-125a, miR-125b-1, miR-146a, and miR-127
3	[6]	Microarray	miR-20a, miR-21, miR-214, miR-92-2, miR-107, miR-128b, miR-181b-1, miR-199a-1, miR-223, miR-24-1, miR-24-2, miR-25, miR-29b-2, miR-30c, miR-32, miR-17-5p, miR-221, miR-106a, miR-146a, and miR-191
4	[23]	Northern blot, qRT-PCR	miR-21
5	[5]	Northern blot, qRT-PCR	miR-21, miR-107, and miR-103
6	[24]	Northern blot, qRT-PCR	miR-155
7	[25]	Northern blot, qRT-PCR	miR-107
8	[26]	Northern blot, qRT-PCR	miR-21
9	[27]	Northern blot, qRT-PCR	miR-155, miR-203, miR-210, and miR-222
10	[28]	Northern blot, qRT-PCR	miR-21 and miR-221
11	[29]	Northern blot, qRT-PCR	miR-10a
12	[30]	qRT-PCR, luciferase assay	miR-214

**Table 2:** Genes in association with pancreatic cancer are shown in the network.

Number	Target gene	Malacard score	microRNA	*P* value
1	ARL4C	16.286	miR-181b	0.01238
2	CCR9	12.916	miR-24	0.00077
3	ACVR2B	12.855	miR-181b	0.01174
		12.855	miR-103	0.01831
		12.855	miR-210	0.02103
		12.855	miR-107	0.02811
4	SMAD2	12.814	miR-24	0.02287
5	ABCC5	12.387	miR-210	0.0 866
6	GFRA1	12.056	miR-221	0.01184
		12.056	miR-210	0.04776
7	CASP2	11.982	miR-24	0.02298
8	PTPN11	10.469	miR-24	0.0054
9	PPARA	10.283	miR-222	0.04031
10	CD34	10.128	miR-24	0.00764
11	PGR	9.519	miR-24	0.02159
12	LLGL1	8.843	miR-24	0.01736
13	MTHFR	8.68	miR-103	0.04494
		8.68	miR-107	0.03749
14	SLC36A1	8.592	miR-24	0.01746
15	SPNS2	8.015	miR-24	0.02632
16	TGFBR2	7.987	miR-181d	0.03812
17	PODXL	7.57	miR-221	0.01018
18	HIPK2	7.531	miR-24	0.02163
19	GNE	7.457	miR-24	0.01318
20	TCF3	7.151	miR-24	0.01106

**Table 3:** Genes target that are regulated with more than one microRNA and are displayed in the network.

Number	Target gene	Description	GO annotation	microRNAs	*P* value
1	ACVR2B*	Active in A receptor, type IIB	Muscle growth	hsa-miR-181b hsa-miR-103 hsa-miR-210 hsa-miR-107	0.011748 0.018318 0.021035 0.028112

2	AP3S2	Adaptor-related protein complex 3, sigma 2 subunit	Protein transporter activity	hsa-miR-24 hsa-miR-210	0.000145 0.010804

3	C22orf29	Chromosome 22 open reading frame 29	Protein binding; play a key role in apoptotic signaling activity	hsa-miR-103 hsa-miR-107	0.001947 0.00305

4	C22orf46	Chromosome 22 open reading frame 46	Transcription regulation	hsa-miR-221 hsa-miR-181d hsa-miR-181b	0.000716 0.002848 0.003748

5	DYRK2	Dual-specificity tyrosine-(Y)-phosphorylation regulated kinase 2	Translation insulin regulation of translation and DNA damage	hsa-miR-103 hsa-miR-210	0.024406 0.027992

6	GFRA1*	GDNF family receptor alpha 1; development of EGFR signaling pathway and axon guidance	Integrin binding and receptor binding	hsa-miR-221 hsa-miR-210	0.011847 0.04776

7	GPR107	G protein-coupled receptor 107		hsa-miR-222 hsa-miR-221	0.012316 0.037447

8	HOOK3	Hook microtubule-tethering protein 3	Attach to microtubule, and more divergent C-terminal domains, which mediate binding to organelles	hsa-miR-222 hsa-miR-221 hsa-miR-181b	0.000872 0.002815 0.014215

9	IRGQ	Immunity-related GTPase family, Q		hsa-miR-24 hsa-miR-222	0.011954 0.027394

10	KSR2	Kinase suppressor of ras 2	Protein serine/threonine kinase activity and MAP-kinase scaffold activity	hsa-miR-24 hsa-miR-222	0.030872 0.048884

11	MTHFR*	Methylenetetrahydrofolate reductase (NAD(P)H)	Methylenetetrahydrofolate reductase (NADPH) activity and modified amino acid binding	hsa-miR-103 hsa-miR-107	0.044941 0.03749

12	N4BP1	NEDD4 binding protein 1	Protein binding	hsa-miR-222 hsa-miR-221	0.001158 0.003722

13	NXF1	Nuclear RNA export factor 1	Nucleocytoplasmic transporter activity and nucleotide binding mRNA processing and transport of mature mRNAs derived from intronless transcripts	hsa-miR-24 hsa-miR-31	0.005296 0.046764

14	PGPEP1	Pyroglutamyl-peptidase I	Cysteine-type peptidase activity	hsa-miR-31	0.000891
				hsa-miR-210	0.003034

15	PLEKHM3	Pleckstrin homology domain containing, family M, and member 3	Phospholipid binding and metal ion binding	hsa-miR-222	0.005244
				hsa-miR-221	0.016343
				hsa-miR-146a	0.046599

16	TSPAN14	Tetraspanin 14		hsa-miR-24	0.000255
				hsa-miR-210	0.01834

17	UBN2	Ubinuclein 2		hsa-miR-24	0.005656
				hsa-miR-222	0.013163

18	ZBTB42	Zinc finger and BTB domain containing 42	DNA binding and zinc ion binding transcriptional repressor	hsa-miR-181d hsa-miR-181b	0.025341 0.032903

*Target gene that are common on Tables 2 and 3.

**Table 4:** GO: discovered categories for the target genes that participate in microRNAs-mRNAs interactions in pancreatic cancer.

Attribute	Descriptor	Number of genes	*P* value
Phenotype	Mortality/aging	50	2.71*E* − 07
	Growth/size phenotype	42	4.43*E* − 07
	Behavior/neurological phenotype	36	8.64*E* − 07

GO: molecular function	Protein binding	63	3.58*E* − 07
	Sequence-specific DNA binding transcription factor activity	19	2.01*E* − 06
	Zinc ion binding	20	3.56*E* − 05

GO: cellular component	Cytoplasm	56	1.43*E* − 09
	Nucleolus	30	1.03*E* − 08
	Nucleus	59	4.66*E* − 08

GO: biological process	Transcription, DNA-dependent	32	2.34*E* − 08
	Smoothened signaling pathway	5	1.72*E* − 05

Domain	Ser/Thr_kinase_AS	11	3.29*E* − 06
	PHF	6	7.42*E* − 06
	Prot_kinase_cat_dom	13	8.79*E* − 06
	Znf_PHD-finger	6	1.48*E* − 05
	Kinase-like_dom	13	2.03*E* − 05
	Znf_PHD	6	2.73*E* − 05
